# BioID-based proteomic analysis of the Bid interactome identifies novel proteins involved in cell-cycle-dependent apoptotic priming

**DOI:** 10.1038/s41419-020-03091-8

**Published:** 2020-10-16

**Authors:** Robert Pedley, Louise E. King, Venkatesh Mallikarjun, Pengbo Wang, Joe Swift, Keith Brennan, Andrew P. Gilmore

**Affiliations:** 1 Wellcome Centre for Cell-Matrix Research, Faculty of Biology, Medicine and Health, Manchester Academic Health Science Centre, University of Manchester, Manchester, UK; 2 Division of Cancer Sciences, Faculty of Biology, Medicine and Health, Manchester Academic Health Science Centre, University of Manchester, Manchester, UK; 3 Division of Cell Matrix Biology and Regenerative Medicine, Faculty of Biology, Medicine and Health, Manchester Academic Health Science Centre, University of Manchester, Manchester, UK; 4 Present Address: Cancer Research UK Manchester Institute, Faculty of Biology, Medicine and Health, Manchester Academic Health Science Centre, University of Manchester, Manchester, UK

**Keywords:** Apoptosis, Protein-protein interaction networks

## Abstract

Apoptotic priming controls the commitment of cells to apoptosis by determining how close they lie to mitochondrial permeabilisation. Variations in priming are important for how both healthy and cancer cells respond to chemotherapeutic agents, but how it is dynamically coordinated by Bcl-2 proteins remains unclear. The Bcl-2 family protein Bid is phosphorylated when cells enter mitosis, increasing apoptotic priming and sensitivity to antimitotic drugs. Here, we report an unbiased proximity biotinylation (BioID) screen to identify regulators of apoptotic priming in mitosis, using Bid as bait. The screen primarily identified proteins outside of the canonical Bid interactome. Specifically, we found that voltage-dependent anion-selective channel protein 2 (VDAC2) was required for Bid phosphorylation-dependent changes in apoptotic priming during mitosis. These results highlight the importance of the wider Bcl-2 family interactome in regulating the temporal control of apoptotic priming.

## Introduction

Cells respond to an apoptotic stimulus via mitochondrial outer-membrane permeabilisation (MOMP), releasing pro-apoptotic factors into the cytosol^1^. However, the proportion of cells that undergo MOMP varies both within and between different cell types^2–4^. Apoptotic priming refers to how close a cell lies to MOMP^5,6^. Primed cells readily undergo MOMP following a pro-apoptotic signal, whereas unprimed cells tolerate a greater level of damage. Apoptotic priming of cancer cells can predict the response to chemotherapy^7,8^, and changes dynamically as cells respond to damage signals. How these changes are coordinated is poorly understood^6,9^.

MOMP is controlled by Bcl-2 family proteins, comprising pro-apoptotic Bax and Bak, which form the pores that drive MOMP, anti-apoptotic proteins, like Bcl-XL, Bcl-2 and Mcl-1, that suppress MOMP by sequestering pro-apoptotic proteins and BH3-only proteins, which regulate the activity of the other two groups^10,11^. BH3-only proteins may be activators or sensitisers. Activators, like Bim and Bid, bind Bax and Bak to initiate MOMP. These activators can be sequestered by anti-apoptotic Bcl-2 proteins, but then released by sensitisers, such as Bad^12^. Thus, variation in the expression and interaction landscape of the Bcl-2 family sets the threshold for MOMP, with changes in this balance having profound consequences^13^.

Cells become primed during mitosis, with those failing to divide being eliminated^9^. Priming is reduced once cells satisfy the spindle-assembly checkpoint (SAC) and enter anaphase. Drugs like Taxol exploit this by preventing transition to anaphase, but a gradual degradation of cyclin B1 can allow cancer cells to exit mitosis without satisfying the SAC, termed mitotic slippage^14^. Mitosis has been linked to post-translational modification of several Bcl-2 proteins^15–20^, changes in their expression^21,22^ and degradation^16,23^. We previously demonstrated that Bid, an activator BH3-only protein, becomes phosphorylated in mitosis, which increased apoptotic priming independent of its canonical regulation via caspase-8 proteolysis^20^. Together, these events set the mitotic Bcl-2 landscape and therefore determine how long a cell resists apoptosis whilst attempting to satisfy the SAC. Thus, mitosis provides an opportunity for understanding how cells dynamically adjust priming within precise temporal boundaries.

To identify how cells coordinate temporal changes in priming during mitosis, we performed an unbiased proteomic screen using proximity-dependent biotin identification (BioID)^24^. As we previously identified that Bid phosphorylation regulates priming in mitotic cells, we used full-length (FL) Bid as bait. We identify that the predominant proteins identified in live cells are outside the canonical Bid interactome and the Bcl-2 protein family. In particular, we identify that the outer mitochondrial porin, voltage-dependent anion channel 2 (VDAC2), is required for cells to temporally increase apoptotic priming following mitotic Bid phosphorylation.

## Results

### Using BioID to interrogate the Bid interactome in live cells

In order to interrogate temporal regulation of priming, we examined post-translational modifications (PTMs) known to alter Bcl-2 protein function in mitosis. Focusing on mitosis is useful for a proteomic screen targeting temporal changes in priming, as cells are readily enriched in the M phase. Bid becomes phosphorylated in mitosis (serine 66 in mouse, 67 in human; mBid and hBid, respectively)^20^, which increased priming in colon carcinoma cells and their sensitivity to apoptosis during Taxol-induced mitotic arrest. We decided to exploit this temporal regulation of Bid phosphorylation to identify mitosis-specific regulators of priming.

**Fig. 1:**
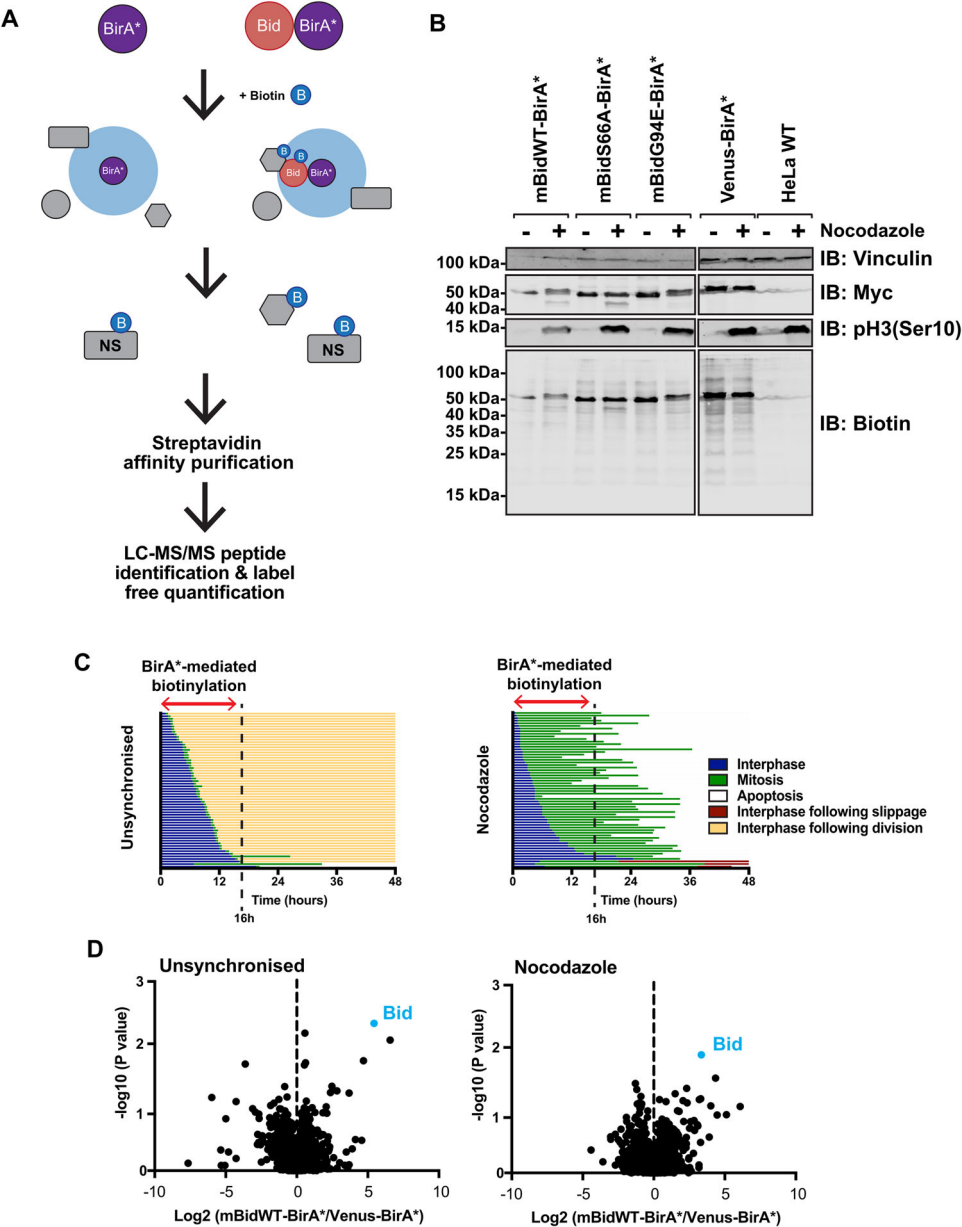
BioID workflow for identification of Bid vicinal proteins in mitotic cells. **A** Schematic diagram of BioID labelling strategy. Selective biotinylation of proximal proteins is followed by stringent cell lysis for streptavidin-affinity purification and identification/quantification by LC–MS/MS. **B** HeLa cells, either wild type or stably expressing mBidWT–BirA*, mBidS66A–BirA*, mBidG94E–BirA* or venus BirA*, were grown with 50 µM biotin for 16 h in the presence (+) or absence (−) of nocodazole. Whole-cell lysates were prepared and examined by immunoblotting for the indicated antibodies and streptavidin. **C** Single-cell-fate profiles of HeLa cells in the presence or absence of nocodazole, imaged over 48 h. Each individual horizontal line represents a single cell. Data represent 90 cells tracked over three independent repeats. A biotin-labelling window of 16 h significantly enriched for mitotic cells compared to untreated controls, without significant enrichment for apoptotic cells. **D** Volcano plot of mean- fold change of biotinylated protein abundance for mBidWT–BirA* vs. venus-BirA* control for unsynchronised and nocodazole-treated samples. Positive ratio indicates enrichment in BidWT sample. *P* value calculated via ANOVA from three independent replicates.

To identify regulators of Bid function, we employed biotin-labelling identification (BioID), a powerful approach for interrogating protein complexes in live cells^24–27^. In this approach, a bait protein is expressed as a fusion with the *E. coli* biotin ligase BirA containing a R118G substitution, along with a myc epitope (BirA*). On addition of excess biotin to the culture media, BirA* generates reactive biotinyl-5′-AMP that can covalently bond to primary amines. The half-life of reactive biotinyl-5′-AMP limits the effective labelling radius to ~20 nm^24^. Consequently, if BirA* is expressed fused to a bait protein, there will be enrichment for biotinylated proteins proximal to that bait, which can be isolated by streptavidin-affinity purification (Fig. 1A). As labelling occurs in situ, BioID is particularly useful for interrogating complexes unsuited to detergent extraction, a known issue for Bcl-2 proteins^28,29^. We therefore generated mBid–BirA* baits to identify interacting partners in live cells.

To validate mBid–BirA* fusions, we compared the pro-apoptotic function of truncated Bid (tBid) fused to either eYFP or BirA*. Both induced similar levels of apoptosis when transiently expressed in HEK-293T cells (Supplementary Fig. S1A). A venus-BirA* fusion did not induce apoptosis. We next validated tBid–BirA*-dependent biotin labelling. Due to its potent pro-apoptotic activity, cells stably expressing tBid–BirA* could not be generated. Therefore, HEK-293T cells transiently expressing either tBid–BirA* or BirA* were grown in media with or without supplementary biotin for 18 h. Whole-cell lysates (WCL) were probed with streptavidin (to detect biotinylated proteins) or anti-myc (Supplementary Fig. S1B). Both BirA*-fusion proteins showed self-labelling in the presence of biotin, although due to the pro-apoptotic activity of tBid–BirA* (Supplementary Fig S1A), labelling appeared weaker than with control BirA*. As expected, levels of the four endogenously biotinylated carboxylases in mammalian cells were unaffected by BirA* or tBid–BirA* expression^30^. To determine if tBid–BirA* could biotinylate known binding partners, we transiently expressed GFP-Bcl-XL in HEK-293T cells, alone or with tBid–BirA*. Transfected cells were supplemented with biotin; WCL was subjected to streptavidin-affinity purification and blotted for GFP and biotin (using anti-GFP and streptavidin, respectively) (Supplementary Fig. S1C). GFP-Bcl-XL bound streptavidin beads only when co-expressed with tBid–BirA*, confirming that BioID captured interactions between tBid and anti-apoptotic Bcl-2 proteins in situ. Finally, we visualised biotin in cells expressing tBid–BirA* or BirA* using immunofluorescence microscopy (Supplementary Fig. S1D). Only cells expressing either tBid–BirA* or BirA* were positive for biotin. In tBid–BirA*-expressing cells, both the anti-myc and biotin labelling appeared punctate.

**Fig. 2:**
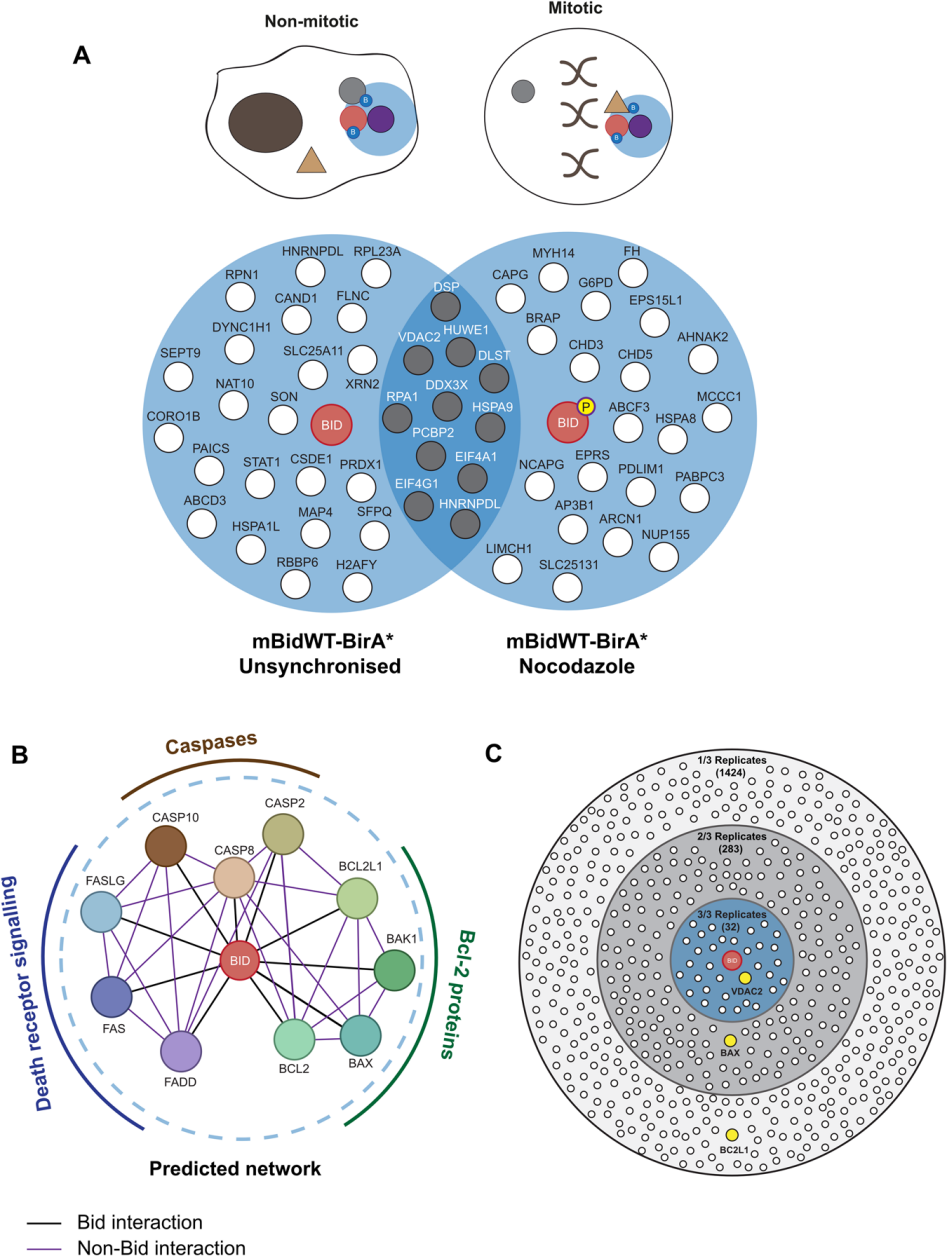
FL-Bid-based BioID mass-spectrometry screen does not enrich canonical Bcl-2 family proteins. **A** Venn diagram showing proteins identified as enriched by mBidWT–BirA* vs. venus BirA* under unsynchronised and nocodazole-treated conditions. Only proteins identified as enriched in all three independent experiments are shown. Proteins identified in both conditions are shaded grey. **B** The predicted protein-interaction network for Bid, derived from the top ten highest-scoring predicted interaction partners as described by STRING. STRING score derived from experimentally validated interactions and interactions in curated databases only. Nodes in blue represent death receptors/death-receptor ligands, nodes in brown represent caspases, while nodes in green represent Bcl-2 proteins. **C** Bcl-2 family proteins are identified in the mBid–BirA* data set when the stringency is lowered. In total, 32 proteins, including voltage-dependent anion channel 2 (VDAC2), were identified as being enriched in mBid–BirA* vs. venus BirA* in three out of three replicates (blue inner circle). Bax was enriched in two out of three replicates. Bcl-XL (BCL2L1) was enriched in one out of three replicates.

To identify mitosis-specific interactions, we used quantitative, label-free mass spectrometry with HeLa cells stably expressing BirA* bait proteins, which allowed robust mitotic enrichment. We generated stable HeLa lines expressing mBidWT–BirA* or venus BirA*, as well as Bid variants that were either not phosphorylated in mitosis (mBidS66A–BirA*), or where the BH3 domain required for interacting with other Bcl-2 proteins was non-functional (mBidG94E–BirA*). Fusion proteins were expressed from a lentivirus co-expressing tagBFP downstream of a T2A-cleavage sequence, allowing FACS selection of cells with similar expression levels of each bait and control (Fig. 1B). Cells were maintained in biotin-supplemented media, containing either nocodazole or DMSO (unsynchronised), for 16 h, which allowed enrichment for labelling in mitosis vs. interphase (Fig. 1C). Labelled proteins were isolated by streptavidin-affinity purification (AP), analysed by LC–MS/MS and abundance quantified using Progenesis QI. Three independent experiments typically identified >400 proteins within each sample (Fig. 1D). Quantification of enrichment for individual proteins was determined by comparing their relative abundance in each mBid–BirA* sample to the experimentally paired venus-BirA* control. To prioritise proteins for further analysis, selection was restricted to those enriched in mBidWT–BirA* cells in all three experimental repeats (Fig. 2A and Tables 1 and 2).

**Table 1:** Summary of enrichment data in unsynchronised HeLa cells, from three independent experiments (R1, R2 and R3) comparing mBidWT–BirA* relative to venus BirA*.

Gene name	Description/protein name	CRAPome %	Sequence coverage	Log2 (ratio)			
				R1	R2	R3	Mean
***BID***	BH3-interacting domain death agonist	1.46	N/A	3.094	3.709	5.261	4.021
*DDX3X*	ATP-dependent RNA helicase DDX3X	51.58	25.0%	0.595	1.327	1.894	1.272
*HNRNPDL*	Heterogeneous nuclear ribonucleoprotein D-like	33.82	6.3%	0.362	0.411	2.660	1.144
*EIF4G1*	Eukaryotic translation-initiation factor 4 gamma 1	24.82	14.0%	2.489	0.249	0.646	1.128
*FLNC*	Filamin C	35.77	4.4%	0.016	0.541	2.190	0.916
*EIF4A1*	Eukaryotic initiation factor 4A-I	46.23	45.0%	0.262	1.085	1.327	0.891
*CAND1*	Cullin-associated NEDD8-dissociated protein 1	21.41	6.3%	0.157	1.904	0.550	0.870
*XRN2*	5′–3′ exoribonuclease 2	23.60	2.9%	0.568	0.379	1.413	0.787
*SEPT9*	Septin 9	19.22	35.0%	0.309	1.482	0.448	0.746
*SLC25A11*	Mitochondrial 2-oxoglutarate/malate carrier protein (fragment)	13.38	8.8%	0.073	0.757	1.389	0.739
*SON*	Protein SON	17.27	0.7%	0.069	0.382	1.564	0.672
*RPA1*	Replication protein A 70-kDa DNA-binding subunit	20.44	8.6%	0.233	0.440	1.328	0.667
*RPN1*	Dolichyl-diphosphooligosaccharide–protein glycosyltransferase subunit 1	22.14	18.0%	1.114	0.428	0.374	0.639
*DSP*	Desmoplakin	30.66	17.0%	0.508	0.772	0.537	0.606
*NAT10*	RNA cytidine acetyltransferase	17.03	4.5%	0.091	0.640	1.053	0.595
***VDAC2***	Voltage-dependent anion channel 2	14.11	49.0%	0.193	1.361	0.143	0.566
*HUWE1*	HECT, UBA and WWE domain-containing 1	18.00	0.3%	0.349	0.087	1.176	0.537
*RBBP6*	E3 ubiquitin–protein ligase RBBP6	9.49	1.9%	0.583	0.313	0.686	0.527
*H2AFY*	Histone H2A	16.55	5.9%	0.770	0.304	0.378	0.484
*SFPQ*	Splicing factor, proline- and glutamine-rich	48.91	7.2%	0.070	0.642	0.659	0.457
*DYNC1H1*	Cytoplasmic dynein 1 heavy chain 1	31.87	3.1%	0.081	0.388	0.873	0.447
*RPL23A*	60s ribosomal protein L23a (fragment)	54.50	20.0%	0.091	0.608	0.596	0.432
*MAP4*	Microtubule-associated protein	20.44	11.0%	0.345	0.767	0.165	0.426
*STAT1*	Signal transducer and activator of transcription 1-alpha/beta	5.60	12.0%	0.056	0.231	0.825	0.371
*HSPA9*	Stress-70 protein, mitochondrial (fragment)	74.70	25.0%	0.774	0.060	0.196	0.343
*PCBP2*	Poly(rC)-binding protein 2 (fragment)	45.99	26.0%	0.125	0.737	0.059	0.307
*DLST*	Dihydrolipoamide S succinyltransferase (E2 component of 2-oxo-glutarate complex)	13.87	4.4%	0.150	0.205	0.532	0.296
*ABCD3*	ATP-binding cassette subfamily D member 3	9.73	3.5%	0.003	0.281	0.567	0.284
*DKFZp686L20222*	Putative uncharacterised protein DKFZp686L20222	19.71	26.0%	0.265	0.289	0.291	0.282
*CORO1B*	Coronin	9.25	38.0%	0.011	0.416	0.336	0.254
*DNA helicase*	DNA helicase	N/A	11.0%	0.274	0.217	0.047	0.179
*PAICS*	Multifunctional protein ADE2	25.79	40.0%	0.101	0.121	0.268	0.164
*PRDX1*	Peroxiredoxin 1	61.56	45.0%	0.057	0.019	0.292	0.122
*CSDE1*	Cold-shock domain-containing E1, RNA-binding isoform	16.30	21.0%	0.064	0.241	0.021	0.109
*HSPA1L*	Heat-shock 70-kDa protein 1-like	94.65	43.0%	1.040	0.037	0.494	0.524

Proteins are ordered by mean enrichment (Log2 ratio mBidWT–BirA* vs. venus BirA*). Only proteins enriched in all three repeats are shown. Those highlighted in italic with an underline have *CRAPome scores >20%. VDAC2* is highlighted in bold italic with an underline. Bid (highlighted in bold italic) is the mouse protein used as bait.

**Table 2:** Summary of enrichment data in nocodazole-treated HeLa cells, from three independent experiments (R1, R2 and R3) comparing mBidWT–BirA* relative to venus BirA*.

				Log2(ratio)			
Gene name	Description/protein name	CRAPome%	Sequence coverage	R1	R2	R3	Mean
***BID***	BH3-interacting domain death agonist (mouse)	1.46	N/A	2.416	2.728	4.072	3.072
*RPA1*	Replication protein A 70-kDa DNA-binding subunit	20.44	2.1%	0.179	0.228	4.966	1.791
*G6PD*	Glucose-6-phosphate 1 dehydrogenase	5.11	5.2%	0.446	0.003	4.326	1.592
*FH*	Fumarate hydratase isoform 1 (fragment)	5.84	8.0%	0.326	1.219	2.902	1.482
*BRAP*	BRCA1-associated protein	4.62	2.2%	0.462	0.242	3.442	1.382
*DDX3X*	ATP-dependent RNA helicase DDX3X	51.58	15.0%	0.449	0.696	2.668	1.271
*MYH14*	Myosin 14	32.12	37.0%	1.025	0.099	2.656	1.260
*CAPG*	Macrophage-capping protein	1.22	16.0%	1.056	0.061	2.014	1.044
*NCAPG*	Non-SMC condensin I complex, subunit G	14.36	2.8%	0.822	0.094	2.107	1.007
***VDAC2***	Voltage-dependent anion channel 2	14.11	35%	0.812	0.914	1.154	0.960
*LIMCH1*	LIM and calponin homology domain-containing protein 1 (fragment)	5.35	2.4%	0.068	0.189	2.532	0.930
*SLC25A31*	ADP/ATP translocase 4	42.82	37.0%	0.091	0.288	2.392	0.924
*EIF4G1*	Eukaryotic translation-initiation factor 4 gamma 1 (fragment)	24.82	19.0%	0.468	0.371	1.804	0.881
*ARCN1*	Archain 1, isoform CRA_b	22.38	9.0%	0.096	0.208	2.311	0.872
*NUP155*	Nucleoporin 155 kDa	11.92	5.5%	0.647	0.066	1.793	0.836
*PCBP2*	Poly(rC)-binding protein 2 (fragment)	45.99	25.0%	0.706	0.299	1.491	0.832
*MCCC1*	Methylcrotonoyl-CoA carboxylase subunit alpha, mitochondrial	21.41	15.0%	0.006	0.113	2.367	0.829
*HSPA9*	Stress-70 protein, mitochondrial (fragment)	74.70	26.0%	1.143	0.040	1.143	0.776
*HSPA8*	Heat-shock cognate 71 kDa of protein (fragment)	96.35	40.0%	0.198	0.250	1.855	0.768
*EIF4A1*	Eukaryotic initiation factor 4A-I (fragment)	46.23	44.0%	0.250	0.842	0.852	0.648
*DLST*	Dihydrolipoamide S succinyltransferase (E2 component of 2-oxo-glutarate complex)	13.87	4.4%	0.138	0.326	1.414	0.626
*CHD3*	Chromodomain-helicase-DNA-binding protein 3	13.63	2.7%	1.232	0.324	0.146	0.567
*HNRNPDL*	Heterogeneous nuclear ribonucleoprotein D-like	33.82	6.3%	0.511	0.327	0.674	0.504
*AP3B1*	Adaptor-related protein complex 3 beta- 1 subunit isoform 1 (fragment)	9.98	3.0%	0.352	0.218	0.849	0.473
*EPRS*	Bifunctional glutamate/proline–tRNA ligase	38.20	25.0%	0.668	0.145	0.568	0.460
*EPS15L1*	Epidermal growth-factor receptor substrate 15-like 1	6.57	15.0%	0.402	0.149	0.830	0.460
*CHD5*	Chromodomain-helicase-DNA-binding protein 5	14.11	1.3%	0.129	0.095	1.137	0.454
*AHNAK2*	Protein AHNAK2	4.87	9.5%	0.339	0.290	0.475	0.368
*DSP*	Desmoplakin	30.66	21.0%	0.132	0.363	0.272	0.256
*PABPC3*	Polyadenylate-binding protein 3	31.63	8.6%	0.121	0.309	0.270	0.233
*ABCF3*	ATP-binding cassette subfamily F member 3 isoform 1 (fragment)	0.73	5.9%	0.143	0.209	0.111	0.154
*HUWE1*	HECT, UBA and WWE domain- containing 1	18.00	0.3%	0.182	0.049	0.184	0.139
*PDLIM1*	PDZ and LIM domain protein 1	7.79	35.0%	0.029	0.185	0.095	0.103

Proteins are ordered by mean enrichment (Log2 ratio mBidWT–BirA* vs. venus BirA*). Only proteins enriched in all three repeats are shown. Those highlighted in italic with an underline have *CRAPome scores >20%. VDAC2* is highlighted in bold italic with an underline. Bid (highlighted in bold italic) is the mouse protein used as bait.

Surprisingly, proteins enriched in both unsynchronised and mitotic mBid–BirA* cells were not associated with Bid’s documented roles within intrinsic or extrinsic apoptotic pathways. The STRING database^31,32^ predicted that the top ten most enriched partners of Bid would be other Bcl-2 proteins, components of the death-induced signalling complex (DISC) and caspases (Fig. 2B). However, proteins enriched by mBid–BirA* did not overlap with this predicted network. We considered that one possibility for this apparent lack of canonical partners might be that these interactions had largely been defined with caspase-cleaved or tBid, and may not represent the main interacting proteins for FL-Bid used in this assay. Alternatively, the structure and/or hydrophobicity of Bcl-2 proteins might mean that these proteins are undetectable by BioID-MS. We therefore asked whether Bcl-2 proteins and/or other STRING-predicted interaction partners were detected in any of the experimental replicates by lowering selection stringency to include proteins enriched in one or two repeats (Fig. 2C). Bax was enriched with mBidWT–BirA* in two repeats, while Bcl-XL was identified in one. Having shown above that tBid–BirA* was capable of biotinylating Bcl-XL in cells (Supplementary Fig. S1C), the inefficient labelling of Bcl-XL and Bax suggested that these proteins were not predominantly in close proximity to FL mBid–BirA* in non-apoptotic cells. As the purpose of the screen was to take an unbiased approach to FL-Bid’s interactions in mitosis, we restricted further analysis to those proteins enriched in all three experimental replicates. Together, these data suggested that Bcl-2 family proteins may not represent the main interaction partners for FL-Bid in live cells.

### VDAC2 is a potential Bid partner in mitosis

**Fig. 3:**
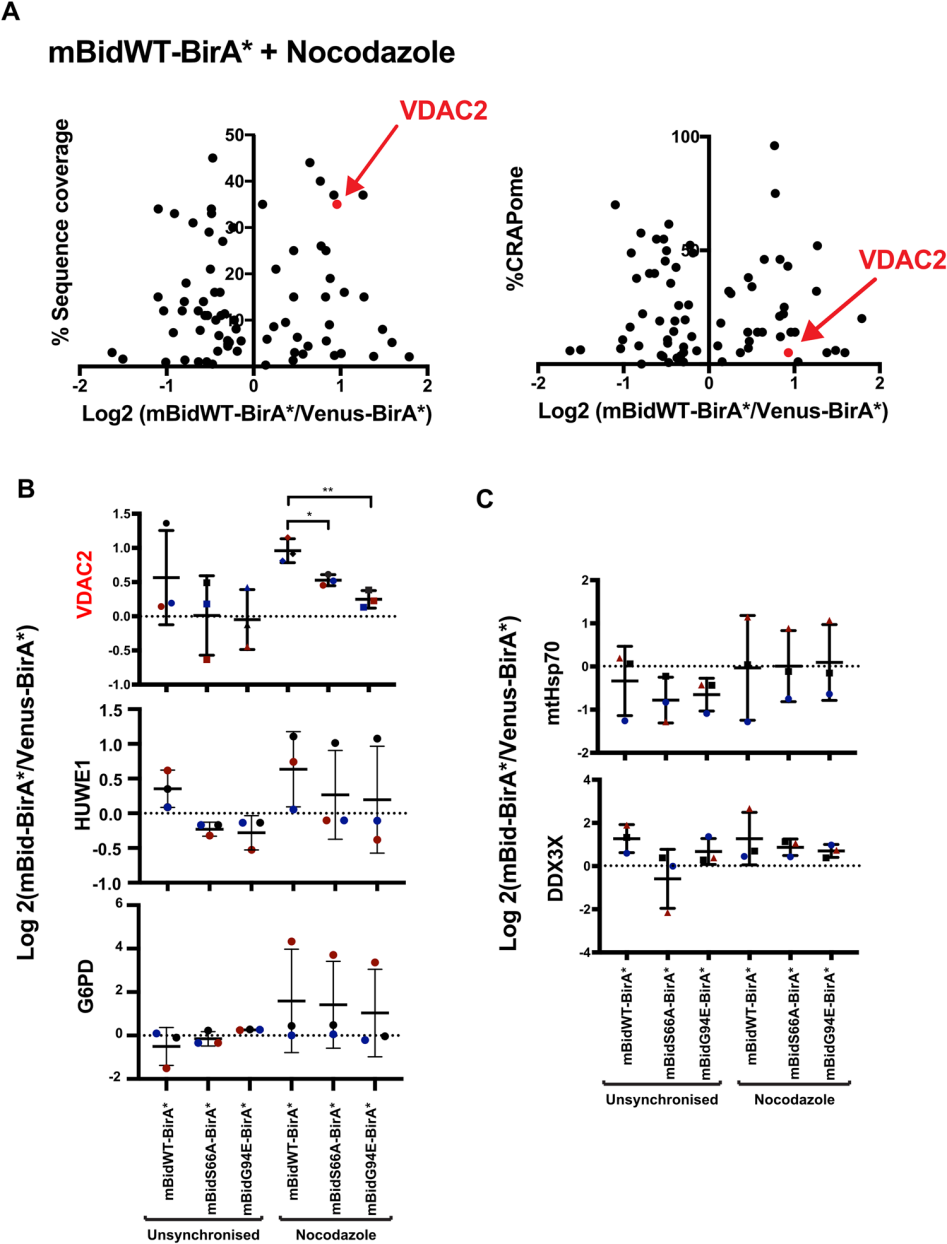
BioID identifies the voltage-dependent anion channel 2, VDAC2, as showing Bid phosphorylation-dependent enrichment in mitotic cells. **A** Scatterplots comparing mean-fold change for mBidWT–BirA* vs. venus BirA* with sequence coverage (left panel) and abundance in published negative-control AP–MS datasets (%CRAPome, right panel). VDAC2 is indicated in both plots as having high sequence coverage, and in low percentage in CRAPome. **B** Summary of MS analysis showing protein-abundant fold change for VDAC2, the HECT domain-containing E3-ubiquitin ligase HUWE1 and glucose-6-phosphate dehydrogenase (G6PD). Data represent mean and SD of three independent replicates. Significance calculated by unpaired *t* tests, *P < 0.05, **P < 0.01. **C** Summary of MS analysis from showing protein-abundant fold change for mtHSP70 and DDX3X, both of which show relatively high scores for their relative frequency in control AP–MS data.

A common issue with AP–MS experiments is detection of background contaminants. Given the absence of predicted Bid-interaction partners, we were concerned that BioID might have predominantly isolated non-specific proteins. To validate candidates, we first compared the sequence coverage of each protein and its relative representation in the contaminant repository for affinity purification (CRAPome), which collates proteins commonly found within negative-control AP–MS datasets (Fig. 3A and Tables 1 and 2)^33^. Falling within the criteria of high sequence coverage and low representation in the CRAPome was the mitochondrial porin, VDAC2. VDAC2 has been extensively linked to Bcl-2 protein-dependent apoptosis^34–37^, despite not being represented within Bid’s STRING-predicted network. Other potential candidate proteins included E3 ubiquitin–protein ligase HUWE1 (MULE/ARF-BP1), reported to regulate Mcl-1, and glucose-6-phosphate dehydrogenase (G6PD), a target of PLK1 in mitosis^38,39^.

To gain confidence for which candidates might associate with Bid in mitosis, we compared their enrichment in unsynchronised and mitotic cells, and between cells expressing mBidWT–BirA*, mBidS66A–BirA* or mBidG94E–BirA* (Fig. 3B). As neither mBidS66A nor mBidG94E increased priming in colon carcinoma cells^20^, we predicted that a specific regulatory partner would not show enrichment with these baits compared to mBidWT. Of potential candidates, only VDAC2 was enriched in mitotic cells expressing the mBidWT–BirA* bait compared to unsynchronised cells. Furthermore, VDAC2 enrichment was significantly lower in mitotic cells expressing either BidS66A–BirA* or BidG94E–BirA* compared to mBidWT–BirA*. Neither HUWE1 nor G6PD showed significant enrichment for specific Bid variants or with mitosis. HUWE1 showed a slight, but not significant, enrichment with mBidWT–BirA* (Fig. 3B). Although we cannot discount either protein being involved in FL-Bid function, we did not pursue them here as the evidence did not support a role in mitosis.

The proteins we identified that are commonly found in control AP–MS datasets showed no specific enrichment either in mitosis or with any of the mBid variants. For example, mitochondrial heat-shock protein 70 (*HSPA9*, mtHsp70) and the ATP-dependent RNA helicase DDX3X showed no difference between lines expressing mBidWT–BirA* or the S66A and G94E variants (Fig. 3C). Notably, VDAC1, which is approximately tenfold more abundant than VDAC2 in HeLa cells^40^, was not enriched in any sample. Importantly, all quantification was performed using only non-conflicting peptides, so sequence similarity between VDAC isoforms was not a confounding issue.

Thus, BioID identified VDAC2 as the principal candidate for a Bid phosphorylation-specific role in mitotic cells.

### VDAC2 couples Bid phosphorylation with increased apoptotic priming in mitosis

As VDAC2 was a potential interaction partner for Bid, we asked whether it and FL-Bid showed similar spatial localisation within the cell. To test this, MCF-7 cells stably expressing mBidWT-GFP were transfected with VDAC2 tagged with a C-terminal V5 epitope (VDAC2-V5). We used VDAC2-V5 as there were no specific antibodies suitable for VDAC2. Previous studies have shown that exogenously expressed VDAC2 with a C-terminal tag is functional and localises to mitochondria, and we observed a similar subcellular distribution^41^ (Supplementary Fig. S2A). Cells expressing mBidWT-GFP and VDAC2-V5 immunostained for GFP and V5 showed that both co-localised in a punctate distribution, suggestive of mitochondria (Supplementary Fig S2B, C).

As co-localisation does not confirm an interaction between proteins, we next sought functional validation. VDAC2 has been linked to both Bax and Bak^34,36,37,42^, and either Bax or Bak is required for Taxol-induced apoptosis^43^. To determine if VDAC2 influenced Taxol-dependent apoptosis, we employed a CRISPR/Cas9-knockout (ko) strategy in cells that showed Bid phosphorylation- dependent priming in mitosis. We initially compared the human mammary carcinoma cell lines, MCF-7 and MDA-MB-231, for susceptibility to apoptosis vs. slippage following treatment with Taxol. Single-cell-fate profiles were obtained through live imaging of unsynchronised cells, in the presence or absence of Taxol, over 65 h, quantifying entry into mitosis, normal division, apoptosis and slippage (Supplementary Fig. S3). Few untreated cells underwent apoptosis. However, in Taxol, MCF-7 cells predominantly underwent apoptosis, whereas MDA-MB-231 cells were prone to slippage (Supplementary Fig. S3D), with inter- and intra-line variation consistent with previous studies^2,44^. We chose MCF-7 cells for generating a VDAC2 ko as these predominantly underwent death in mitosis.

**Fig. 4:**
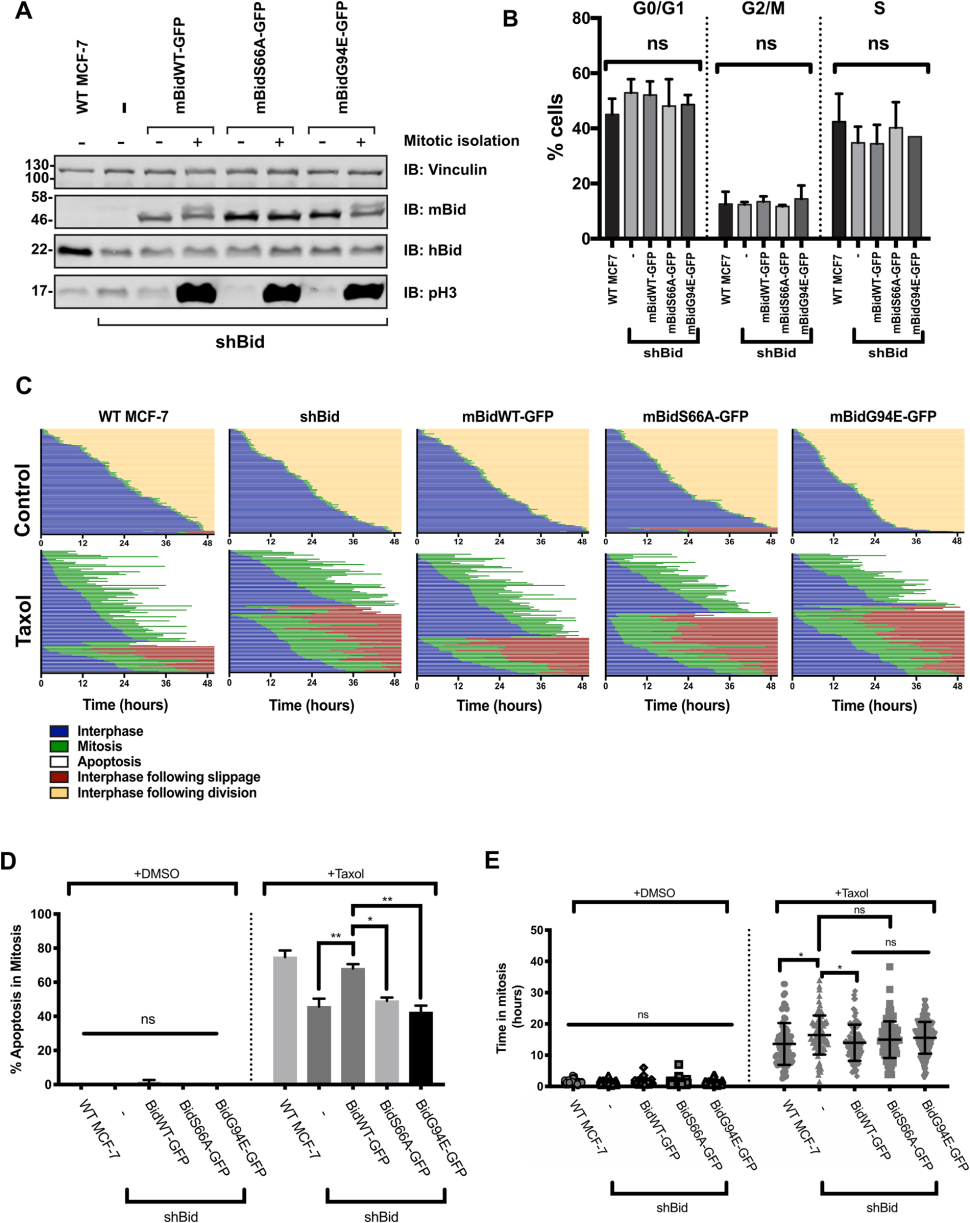
Phosphorylation of Bid regulates apoptotic priming in mitotic breast-cancer cells. **A** MCF-7 cell lines were generated stably expressing either shBid alone or with the indicated mouse Bid-GFP variant (mBidWT-GFP, mBidS66A-GFP and mBidG94E-GFP). Cells were either untreated (−) or enriched for mitosis with 18-h treatment in nocodazole followed by shake off (+). Whole-cell lysates were immunoblotted with the indicated antibodies. Anti-vinculin serves as loading control, while anti-phospho-histone H3 (pH 3) indicates mitosis. **B** Cell-cycle distribution for the MCF-7 lines in **A**, quantified through DAPI staining and flow cytometry. Data were obtained using unsynchronised cells. Data represent mean and SD of 2 independent repeats. **C** Single-cell-fate profiles of the MCF-7 lines in **A**, untreated control or treated with 1 μM Taxol over 50 h. Data represent 90 cells tracked over three independent experiments. **D** Summary of apoptosis in mitosis for the data in **C** for the indicated MCF-7 cell lines. Mean and SD are shown. Data were analysed by one-way ANOVA, followed by Tukey’s multiple-comparison test (ns = non-significant; ***P < 0.001; ****P < 0.0001). **E** Summary of the duration of mitosis, all fates included, for the data in **C**. Mean and SD plotted. Data analysed by one-way ANOVA, followed by Tukey’s multiple- comparison test (ns = non-significant; ***P < 0.001; ****P < 0.0001).

To first confirm that Bid phosphorylation set MCF-7 apoptotic priming in mitosis, we stably knocked down endogenous hBid using a previously verified lentiviral shRNA (shBid)^20^. MCF-7 lines were generated expressing either shBid alone, or with mBidWT-GFP, mBidS66A-GFP or mBidG94E-GFP (Fig. 4A). We simultaneously expressed shBid and the mBid-GFP variants, using a ubiquitin promoter to achieve close-to-endogenous expression levels (Supplementary Fig. S4A), and FACS to select similar levels for each line. Immunoblotting for each variant in unsynchronised and mitotic cells indicated that mBidWT-GFP and mBidG94E-GFP were phosphorylated in mitosis, whereas mBidS66A-GFP was not (Fig. 4A). Quantitative immunoblotting showed that endogenous hBid expression was reduced by approximately 70% (Supplementary Fig. S4A).

Flow cytometry showed that Bid expression, phosphorylation or apoptotic function did not affect normal cell-cycle progression (Fig. 4B). We compared single-cell fates for parental MCF-7 (WT MCF-7) cells with those expressing shBid, alone or with each mBid-GFP variant. In the absence of Taxol, there was no significant apoptosis or slippage in any of the lines (Fig. 4C, D). However, in the presence of Taxol, Bid knockdown significantly shifted MCF-7 cell fate towards slippage (Fig. 4C, D). This was corrected by expression of mBidWT-GFP, but not by mBidS66A-GFP or mBidG94E-GFP. Knockdown of hBid or expression of any of the FL-Bid variants had no impact on the time untreated cells spent in mitosis (Fig. 4C, E). Taxol clearly increased the time cells spent in mitosis. Loss of Bid expression slightly increased the time in Taxol-arrested mitosis relative to parental cells, which was accounted for by the increase in time KD cells were able to remain in mitosis before inducing apoptosis, but not slippage (Supplementary Fig. S4B). Thus, Bid phosphorylation and pro-apoptotic function were required for increasing priming in MCF-7 cells during mitosis. The level of Taxol-induced apoptosis in all the shBid MCF-7 lines could be reset to that of WT MCF-7 cells by treating with the BH3 mimetic, ABT737, indicating that the effect of Bid phosphorylation was to set the level of mitochondrial priming, and suggesting that it was functioning as a sensitiser (Supplementary Fig. S5A, B). ABT737 in the absence of Taxol had no effect on apoptosis. MDA-MB-231 cells stably expressing shBid with mBidWT-GFP, mBidS66A-GFP or mBidG94E-GFP were also examined (Supplementary Fig. S5C, D). As with MCF-7 cells, the Bid variants by themselves did not alter normal mitosis. Interestingly, although the priming baseline in MDA-MB-231 was higher than for MCF-7, there was still an observable reduction in apoptosis in Taxol for cells expressing BidS66A-GFP or BidG94E-GFP compared with mBidWT-GFP, although this was not significant.

**Fig. 5:**
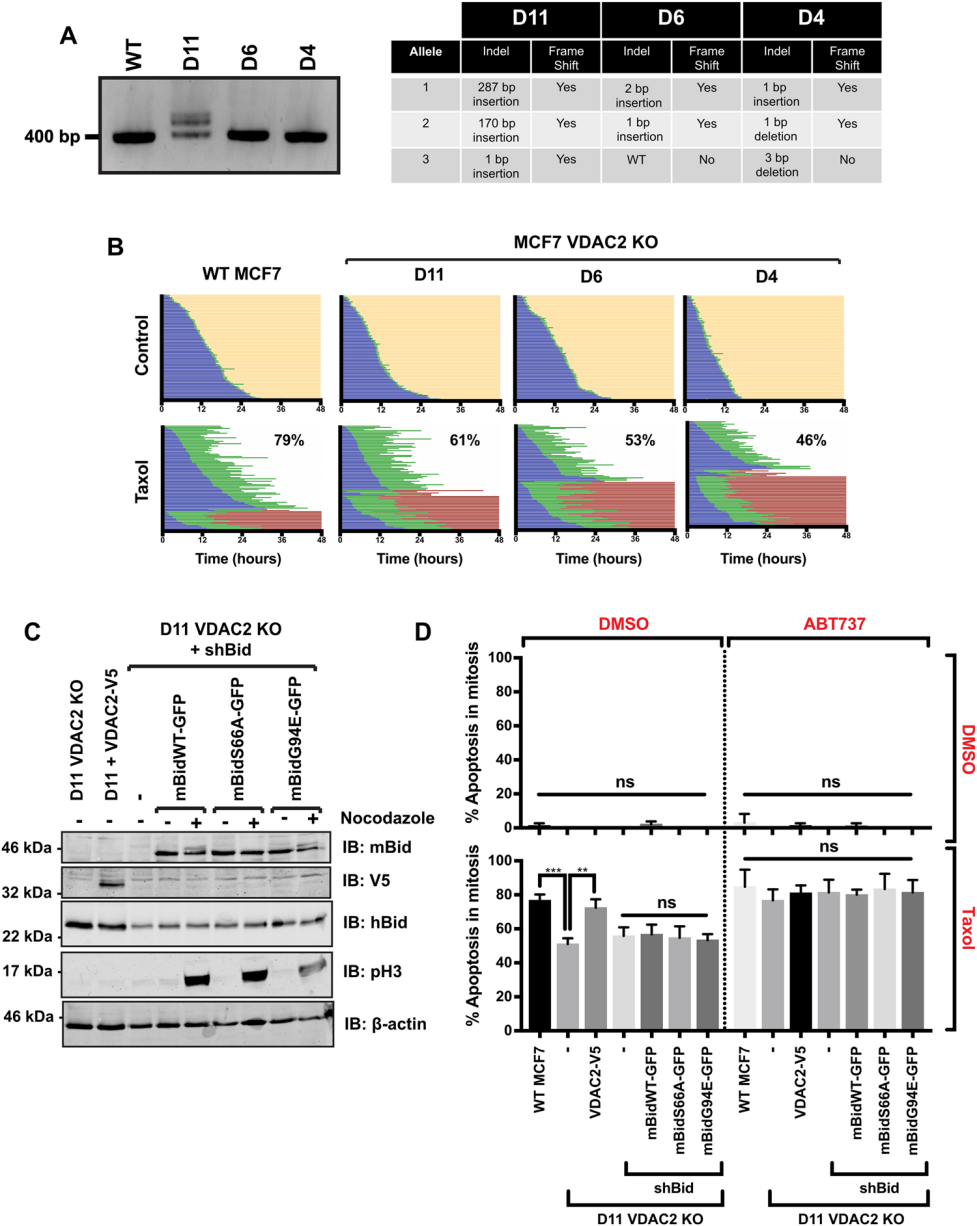
Voltage-dependent anion channel 2 (VDAC2) coordinates Bid phosphorylation-dependent apoptotic priming in mitosis. **A** Single MCF-7 cell clones were grown following CRISPR/Cas9 targeting of *VDAC2*. Left-hand panel—agarose gel electrophoresis of 300-bp PCR product at *VDAC2* CRISPR target PAM site. Right-hand panel—table displaying indels caused by CRISPR/Cas9 determined by direct DNA sequencing of the amplified regions. **B** Single-cell-fate profiles of the three MCF-7 *VDAC2* KO lines in **A**, untreated (control) or treated with 1 μM Taxol over 48 h. Data represent 90 cells over three independent experiments. Percentage of death in mitosis is shown for Taxol-treated cells. **C** D11 *VDAC2* KO MCF-7 cell lines were generated stably expressing VDAC2-V5, shBid alone or shBid in conjunction with the indicated full-length mouse Bid-GFP variants (mBidWT-GFP, mBidS66A-GFP and mBidG94E-GFP). Cells were either untreated or enriched for mitosis with 18-h treatment in nocodazole followed by shake off. Whole-cell lysates were immunoblotted with the indicated antibodies. β-actin serves as loading control, while phospho-histone H3 (pH 3) indicates mitosis. **D** Summary of apoptosis in mitosis for single-cell-fate analysis of the cell lines in **C**, untreated or treated with 1 μM Taxol and/or 5 μM ABT737 over 48 h (single-cell traces in Supplementary Fig. S5C). Data represent 90 cells tracked over three independent experiments. Error bars represent SD. Data analysed by one-way ANOVA, followed by Tukey’s multiple-comparison test. ns = non-significant, **P < 0.01, ***P < 0.001.

As MCF-7 cells showed Bid phosphorylation-dependent priming in mitosis, we targeted the start of the protein-coding region of the *VDAC2* gene in them using CRISPR/Cas9. Three independent clones containing indels were identified, termed D11, D6 and D4 (Fig. 5A). MCF-7 cells have three copies of *VDAC2*. In the absence of a VDAC2-specific antibody, we confirmed VDAC2 deletion by sequencing PCR-amplified regions of the *VDAC2* gene. Sequencing indicated that for D11, all three copies contained frame-shift insertions. D6 had two frame-shift insertions and one WT allele, and D4 two frame-shift insertions and one allele with a 3-bp deletion just after the start of the coding sequence.

We performed cell-fate analysis to compare WT MCF-7 cells to the VDAC2 ko lines (Fig. 5B). There were no differences in normal mitotic progression in untreated cells. However, when treated with Taxol, all three VDAC2 ko lines showed a shift towards slippage compared to WT cells. Furthermore, the proportion of each VDAC2 ko line undergoing slippage was comparable to that seen with shBid MCF-7 cells, or those expressing mBidS66A or mBidG94E (cf. Fig. 4C). Stably expressing VDAC2-V5 (Fig. 5C) in the MCF-7 D11 line restored the level of apoptosis in Taxol to that of WT MCF-7 cells (Fig. 5D and Supplementary Fig. S6A). We compared expression of Bcl-2 family proteins in WT, D11 and D11-VDAC-V5 cells (Supplementary Fig. S6B). As previously shown, VDAC2 ko reduced Bak expression, which was restored in D11-VDAC-V5 cells^42^. There were corresponding increases in Bax and Puma in the D11 cells, but no change in expression of Bcl-XL, Mcl-1, Bim or Bid.

We asked if VDAC2 was required for coupling Bid phosphorylation to priming. The D11 line was stably transduced with shBid alone, or shBid along with mBidWT-GFP, mBidS66A-GFP or mBidG94E-GFP (Fig. 5C). Notably, both mBidWT-GFP and mBidG94E-GFP were still phosphorylated in mitotic D11 cells, seen with the mobility shift in the presence of nocadazole. Cell- fate profiling in the presence or absence of Taxol compared each mBid-GFP D11 line to WT MCF-7, the D11 ko and D11-VDAC2-V5 cells (Supplementary Fig. S6A). Bid knockdown had no additive effect on apoptosis beyond that already brought about by *VDAC2* deletion (Fig. 5D). mBidWT-GFP-, mBidS66A-GFP- or mBidG94E-GFP-expressing D11 cells all showed the same proportion of death vs. slippage in Taxol as the D11-VDAC2 ko (Fig. 5D). Importantly, apoptosis in D11 cells was comparable to shBid MCF-7 cells (cf. Fig. 4C, D). As in Supplementary Fig. S5A, apoptosis following Taxol in all lines was restored to the level of WT MCF-7 cells by treating with ABT737 in combination with Taxol, indicating that VDAC2 deletion did not simply make MCF-7 cells incapable of apoptosis via the intrinsic pathway following delayed mitotic exit. ABT737 by itself did not induce apoptosis in any of the lines, further indicating that cell death is a response to the delay in mitotic exit. These data show that VDAC2 is required to coordinate the changes in apoptotic priming in response to Bid phosphorylation in mitosis.

## Discussion

Cells constantly balance a multitude of pro-survival and pro-death signals. However, it is still unclear how Bcl-2 proteins dynamically adjust this balance in response to rapidly changing circumstances. Using an unbiased proteomic approach to label Bid vicinal proteins, we identified VDAC2 as a coordinator of dynamic apoptotic priming in mitotic cells. As BioID catalyses protein biotin labelling in situ, it allows extraction of integral membrane components without having to maintain direct protein–protein interactions, thus avoiding issues with alterations to Bcl-2 protein interactions in detergents^28^.

Unbiased AP–MS approaches suffer background contaminants, and we used a number of approaches to identify specific interactions. First, by using both BidS66A–BirA* and BidG94E–BirA*, we identified VDAC2 as the only candidate specifically enriched in mitotic cells when Bid was both phosphorylated and had a functional BH3 domain. Thus, VDAC2 enrichment by FL-Bid–BirA* showed a specificity for bait proteins that matched their function in priming mitotic cells. Second, the three mammalian isoforms of VDAC show high sequence similarity, but have distinct roles^45^. Although VDAC1 is tenfold more abundant than VDAC2 in HeLa cells^40^, it was not enriched by any of the mBid–BirA* variants. Third, functional validation through VDAC2 deletion in a cell line (MCF-7) distinct from that used in screen, uncoupled mitotic priming from Bid phosphorylation. Bid was still phosphorylated in mitotic VDAC2 ko cells, indicating that VDAC2 lies downstream of potential Bid kinases. Loss of VDAC2 dampened priming in mitosis to the same extent as seen with Bid knockdown, and itsr combined loss was not additive. Importantly, VDAC2 ko MCF-7 cells were not simply resistant to apoptosis in response to mitotic arrest, as cell death following treatment with Taxol was fully restored with the BH3 mimetic ABT737. Taken together, these data indicate that VDAC2 coordinates FL-Bid functioning as a sensitiser BH3-only protein in this context.

Previous studies have shown that VDAC2 is required for tBid-induced apoptosis in mouse embryonic fibroblasts (MEFs)^41,42^, through recruitment of Bak to mitochondria^46^. Consistent with this, we observed that VDAC2-deficient MCF-7 cells expressed less Bak, although there appeared to be a compensatory increase in Bax. As Bid preferentially activates Bak^47^, together, these findings might suggest a phospho-Bid-VDAC2-Bak axis regulating priming in mitosis. However, the literature on VDAC2 in Bcl-2 protein function is more complicated and contradictory. Originally, VDAC2 was found to inhibit Bak-dependent MOMP, and overexpression of VDAC2 suppressed Bak activation by tBid^34^. A more recent genome-wide CRISPR/Cas9 screen in Mcl-1-deficient MEFs identified VDAC2 as a mediator of Bax-, not Bak-dependent apoptosis^37^. Yet another study found that Bax retrotranslocation from mitochondria was mediated by VDAC2, and that it functioned as a platform to coordinate the interaction between Bax and Bcl-XL^36^. Whilst its role in apoptosis remains unclear, our data support a pivotal role for VDAC2 as a nexus for Bcl-2 protein coordination on the mitochondria, the precise function being context- and stimulus-specific.

The mechanism by which FL-Bid and VDAC2 coordinate apoptotic priming in mitosis remains to be elucidated. phospho-Bid might displace bound Bak or Bax from VDAC2, or bind directly to VDAC2- bound Bak or Bax to trigger activation. However, elucidating the mechanistic details presents some technical issues. Both Bak and VDAC2 are integral mitochondrial membrane proteins, and the denaturing conditions required to extract them would likely not be compatible with conventional co-immunoprecipitation approaches. To this end, the use of approaches such as BioID can provide novel insights.

The lack of canonical Bcl-2 family proteins identified using FL-Bid as our bait was unexpected. Bid was originally identified as an interacting partner of both Bcl-2 and Bax using a phage-expression library^48^. Whilst FL-Bid is pro-apoptotic^49,50^, it has been predominantly studied in the context of cleavage by caspase 8, which converts Bid into a potent direct activator BH3-only protein^51,52^. Thus, interacting partners of Bid defined in the literature and represented in STRING are those associated with its cleaved form in dead or dying cells. tBid–BirA*-labelled GFP-Bcl-XL, and Bcl-XL and Bax peptides were detected in our BioID screen in some experimental replicates. However, these interactions may represent those cells that have committed to apoptosis, whereas in cells that have not yet committed, FL-Bid may associate with proteins outside the Bcl-2 family and extrinsic pathway. Other non-canonical interactions have been identified for BH3-only proteins^53,54^, and using an approach such as BioID will allow interrogation of the Bcl-2 family interactome in a non-apoptotic context. With the advent of Bcl-2 protein-targeted drugs, it is essential to understand the wider interactions of these proteins and the complex layers of regulation, which modulate their function.

**Fig. 6:**
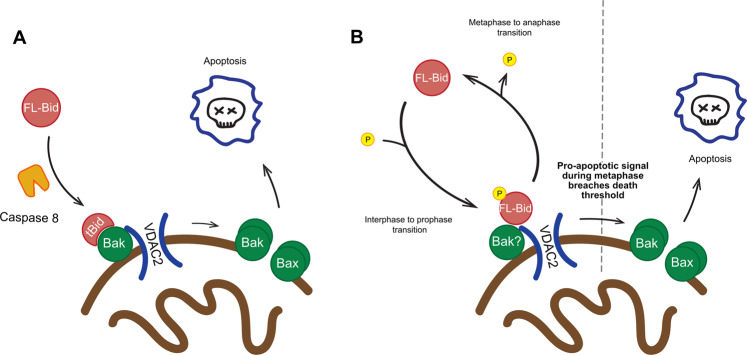
Summary of possible models for voltage-dependent anion channel 2 (VDAC2) regulation of Bid. **A** Caspase-8-cleaved Bid or exogenously expressed tBid acts as a direct activator BH3-only protein. VDAC2 has been shown to interact with Bak and Bax at mitochondria, and this interaction is necessary for their recruitment to the OMM^35,42^. Activation of Bid by caspase 8 then allows tBid to activate mitochondrial bound Bak, resulting in apoptosis commitment and MOMP. In an alternative model, VDAC2 suppresses Bak activation until it is displaced by direct activator BH3 proteins such as tBid^34^. **B** In mitosis, FL-Bid acts as a sensitiser BH3-only protein, shifting apoptotic priming but not driving commitment to MOMP. Phosphorylation of FL-Bid at serine 66 allows its recruitment proximal to VDAC2 and increases priming, but commitment only results if mitotic exit is delayed. Possible mechanisms by which pS66 Bid mediates an increase in priming are through direct interaction with VDAC2, or by interacting with VDAC2-bound proteins, such as Bak. Upon transition to anaphase, Bid is dephosphorylated and priming is reduced.

Finally, these results add to our understanding of how cells coordinate the dynamic changes in priming during mitosis. Mitosis represents a clinically important therapeutic target for drugs like Taxol, and numerous studies have implicated roles for different Bcl-2 family proteins. A genome-wide screen in HeLa cells identified Bax as a key protein for Taxol-mediated cell death, whereas the same approach in colon carcinoma cells did not directly identify any Bcl-2 protein, but instead found a Myc-dependent transcription of Bid, Bim and Noxa^21,22^. An siRNA approach in HeLa cells specifically targeted at Bcl-2 proteins identified roles for Noxa, Bim and Mcl-1^55^. Together, these studies indicate that the outcome of delayed mitotic exit is not determined by a single BH3 protein or multidomain effector, but rather the dynamic interplay of many, the balance of which varies between, and likely within, cell types. Several Bcl-2 proteins undergo mitosis-specific modifications, including Bcl-2, Bcl-XL, Mcl-1, Bid and BimEL, each appearing to have distinct roles^17–20^. However, collectively, they result in the dynamic accumulation of apoptotic priming if anaphase is delayed^9^. Our data now show that, unlike caspase-8 cleavage, phosphorylation of FL-Bid does not convert it into an activator BH3-only protein. Instead, phosphorylated FL-Bid reversibly increases apoptotic priming in mitosis (Fig. 6). It is interesting to speculate that in addition to its established function as a direct activator downstream of death receptors, a significant role for Bid may be as a sensitiser during the cell cycle.

## Methods

### Cell culture

MCF-7, BT-474, MDA-MB-231, SK-BR-3, MCF-10A, HEK-293T and HeLa cells were acquired from ATCC (American Type Culture Collection). MCF-10A cells were grown in DMEM-F12 supplemented with 5% horse serum (v/v), 100 ng/ml cholera toxin, 20 ng/ml epidermal growth factor, 0.5 g/ml hydrocortisone, 10 mg/ml insulin, 100 U/ml penicillin and 100 mg/ml streptomycin. HeLa cells were grown in EMEM supplemented with 10% foetal bovine serum (v/v), 100 U/ml penicillin and 100 mg/ml streptomycin. All other cell lines were cultured in DMEM supplemented with 10% foetal bovine serum (v/v), 100 U/ml penicillin and 100 mg/ml streptomycin.

### Single-cell-fate tracking

In all, 1 µM Taxol (Sigma-Aldrich), 5 µM ABT737 (Sigma-Aldrich) or DMSO diluted in complete growth media was added to cells 30 min prior to imaging. Images were acquired at 15-min intervals for 48–60 h on an AS MDW live-cell imaging system (Leica) in bright field using a ×20 objective and utilising imaging software Image Pro 6.3 (Media Cybernetics Ltd). Point visiting was used to allow multiple positions to be imaged within the same time course, and cells were maintained at 37 °C and 5% CO_2_. Image stacks were analysed in ImageJ (National Institutes of Health), where single cells were identified and followed manually using cellular morphology to determine cell-cycle progression and fate. Cells were described as being in mitosis from the initial point at which a cell began to round, until the first frame of cytokinesis or membrane blebbing. Cells that exhibited morphology associated with apoptosis, such as membrane blebbing, shrinking of the cell body, and in the later stages, the formation of apoptotic bodies or secondary necrosis was described as having undergone apoptosis in mitosis. Cells that exited mitosis as a single cell, or as two or more non-identical daughter cells, were grouped together under the definition of having undergone mitotic slippage.

### Antibodies, immunofluorescence and immunoblotting

The following antibodies were used for immunoblotting and immunoflourescence: rabbit anti-Bid (ProteinTech, # 10988-1-AP), mouse anti-Myc (Millipore, # 05-724), rabbit anti-V5 (Sigma-Aldrich, # V8157), mouse anti-mtHsp70 (Thermo, # MA3-028), rabbit anti-GFP (Invitrogen, # PA1-9533), goat anti-β-actin (Abcam, # Ab8229), rabbit anti-phospho-Histone serine10 (Millipore, # 06-570) and mouse anti-vinculin (Abcam, # Ab9532). To detect biotinylated proteins, we used DyLight 549 Streptavidin (Vector laboratories, # SA-5549) and IRDye680 Strepavidin (LiCor, # 925-68079).

Immunofluorescent staining of cells was performed as previously described^6^, using Alexafluor 594- and 488-conjugated secondary antibodies (Stratech). Immunostained cells were visualised (wide field) using a Zeiss AxioImager M2 florescence microscope with ×63 (NA 1.4) PLAN-APOCHROMAT objective. Images were acquired with Micro-Manager software and processed using ImageJ. For confocal microscopy, images were collected on a Leica TCS SP8 AOBS-inverted confocal using a ×20 Plan Fluotar objective and ×3 confocal zoom. The confocal settings were as follows: pinhole 0.6 airy unit, scan speed 1000-Hz unidirectional. Images were collected sequentially to eliminate crosstalk between channels. When acquiring 3D optical stacks, the confocal software was used to determine the optimal number of *Z* sections. Raw images were then deconvolved using the Huygens Pro software (SVI), and maximum-intensity projections of these deconvolved images are shown in the ‘Results’.

For immunoblotting, proteins were separated by SDS PAGE and transferred to nitrocellulose. Following incubation with primary antibodies, proteins were detected using IrDye 800 and 680 secondary antibodies and an Odyssey CLx imager (LiCor). Quantification of blots was performed using ImageStudio (LiCor).

### Mitotic enrichment of cultured cells

Asynchronous cell populations were treated with nocodazole (Sigma-Aldrich, 200 ng/mL in complete growth media) for 16 h to arrest cells in mitosis. Mitotic cells were then dislodged from the cell-culture dish by tapping the vessel laterally against the bench. Media containing dislodged mitotic cells was removed and centrifuged at 350 RCF for 5 min to pellet cells ready for subsequent analysis.

### Flow-cytometry analysis of the cell cycle

Cell-cycle distribution within a population was quantified by DNA content. Cycling cells were fixed with 70% ethanol and stained with DAPI. Stained cell samples were analysed on a BD Biosciences Fortessa flow cytometer, using Diva Version 8 (BD Biosciences) to collect raw data. Raw data were then analysed using Modfit LT (Version 5, Verity Software House) to quantify the distribution between cell-cycle phases.

### BioID-mediated proximity labelling

For small-scale assays such as western blotting or immunofluorescent staining, cells expressing BirA*-fusion proteins were seeded into a 6-well plate to ~80% confluence on the day of labelling. Growth media was replaced with complete growth media supplemented with 50 µM biotin (Sigma-Aldrich), and cells cultured for a further 16 h. Following incubation, biotin-supplemented media was removed and replaced with regular growth media for 1 h. Cells were then washed three times in PBS and assayed as appropriate.

For mass-spectrometry experiments, cells expressing BioID-fusion proteins were seeded into two 100-mm-diameter dishes to be approximately 80% confluent the next day. On the day of labelling, growth media was replaced with complete growth media supplemented with 50 µM biotin (Sigma-Aldrich) and cultured for a further 16 h, along with any other drug treatment. Following incubation, biotin-supplemented media was removed and replaced with regular growth media for 1 h to allow free biotin to diffuse out of cells. Cells were then washed three times in PBS and lysed in RIPA supplemented with 1× protease inhibitor cocktail (Millipore), 100 mM Na_3_VO_4_ and 500 mM NaF. Following lysis, lysates from both dishes were pooled, protein concentration measured and biotinylated proteins isolated via streptavidin-bead purification.

### Streptavidin-affinity purification

Biotinylated proteins were isolated using MagReSyn^®^ streptavidin (MagReSyn^®^). In total, 100 µL of bead slurry was decanted into a microcentrifuge tube and exposed to a magnetic field to separate the liquid and bead phases. The liquid phase was discarded, and beads were washed three times in ice-cold lysis buffer. Appropriate volumes of cell lysate were added to beads to yield 1 mg of total protein, and the volume made up to a total of 1 mL with lysis buffer. Lysates and beads were incubated overnight at 4 °C on an end-over-end tumbler. The next day, beads were collected, washed twice in lysis buffer, once in urea wash buffer (2 M urea, 10 mM Tris-HCl, pH 8.0), then three more times with lysis buffer. Following the final wash, beads were resuspended in 100 µL of 1× lithium dodecyl sulfate sample buffer (Novex NuPAGE) supplemented with 2 mM biotin and 50 mM dithiothreitol (DTT) and incubated at 95 °C for 5 min to elute bound proteins. Following elution, beads were collected and concentrated to 50 µL in a vacuum concentrator. In all, 45 µL of sample was immediately subjected to SDS PAGE and processed for digestion in preparation for mass spectrometry, while the remaining 5 µL was analysed via western blotting to confirm biotinylation and protein enrichment.

### Mass spectrometry and protein identification

Affinity-purified biotinylated protein samples were briefly separated by SDS PAGE, then fixed and stained using InstantBlue (Expedeon). Gel fragments containing protein samples were digested with trypsin and peptides extracted under standard conditions. Peptides were subjected to liquid chromatography tandem mass spectrometry (LC–MS/MS) using a Thermo Orbitrap Elite coupled with a Thermo nanoRSLC system (Thermo Fisher Scientific). Peptides were selected for fragmentation automatically by data-dependent analysis. Raw data were processed using Progenesis QI (v4.1, Nonlinear Dynamics) and searched against the SwissProt and Trembl databases (accessed July, 2017). Database searches were performed against the human, mouse and *E. coli* proteome databases with tryptic digest specificity, allowing a maximum of two missed cleavages, and an initial mass tolerance of 5 ppm (parts per million). Carbamidomethylation of cysteine was applied as a fixed modification, while N-acylation and oxidation of methionine, as well as the biotinylation of lysine, were applied as variable modifications during the database searches.

### Label-free protein quantification and data analysis

Relative label-free protein quantification of mass-spectrometry experiments was performed using Progenesis QI (v4.1, Nonlinear Dynamics). Raw data were processed and aligned in Progenesis QI. Once aligned, relative protein quantification was calculated from the abundances of ions with three or more isoforms identified as being from unique (non-conflicting) peptides. Comparisons between relative protein abundances were made between proteins isolated from wild-type or mutant Bid–BirA* lysates (sample) and proteins isolated from venus-BirA* lysates (control) to calculate a fold change. Sample and control protein abundances were paired based on drug treatment, e.g., WTBid-BirA* + nocodazole was compared with venus BirA* + nocodazole. Statistical analysis of proteomics data was performed in Progenesis QI using ANOVA.

### STRING analysis

Predicted interaction networks were computed by STRING (Search Tool for the Retrieval of Interacting Genes/Proteins) from the top 10 highest-scoring predicted functional partners for Bid^31,32^. Only data from experimental validated interactions and interactions in curated databases were used to score predicted functional partners. STRING Database v11.0, accessed April 2019.

### VDAC2 deletion by CRISPR/Cas9

Deletion of endogenous *VDAC2* was achieved through CRISPR/Cas9. Two sgRNA sequences (CAATGTGTATTCCTCCATC, GATGGAGGAATACACATTG), designed to target upstream of the second protein-coding exon, were cloned into PX458 (pSpCas9(BB)-2A-GFP). PX458 containing the VDAC2-targeting guides was transfected into low-passage WT MCF-7 cells and sorted by FACS for GFP expression. Single cells were isolated from the sort and plated into individual wells of 96-well plates. Wells in which clonal cell populations had expanded were transferred to larger cultures, and genomic DNA extracted for identification of indels by PCR and sequencing.

### Statistical analysis

Statistical analysis was performed using GraphPad Prism v7. Details of specific tests used, the number of biological replicates and sample size, are given in the figure legends.

## Supplementary information

supplementary figure legends

supplementary figure 1

supplementary figure 2

supplementary figure 3

supplementary figure 4

supplementary figure 5

supplementary figure 6
