# Supplementary material for: BioID-based proteomic analysis of the Bid interactome identifies novel proteins involved in cell-cycle-dependent apoptotic priming
Source: Cell Death Dis. 2020 Oct 16;11(10):872. doi: 10.1038/s41419-020-03091-8 (PMC7567853; doi:10.1038/s41419-020-03091-8)
Supplement: Supplementary file 1 — supplementary figure legends [file 41419_2020_3091_MOESM1_ESM.docx]

**BioID based proteomic analysis of the Bid interactome identifies novel interactions involved in cell cycle dependent apoptotic priming.**

Robert Pedley, Louise E. King, Venkatesh Mallikarjun, Pengbo Wang, Joe Swift, Keith Brennan, and Andrew P. Gilmore.

**Supplementary data.**

**Supplementary figure legends**

**Figure S1.** *Generation and validation of a Bid-BirA* bait fusion protein.*

(A) HEK-293T cells were transiently transfected with plasmids expressing either tBid-eYFP, tBid-BirA* or Venus-BirA*. After an overnight incubation cells were harvested, cytospun onto slides and stained for their respective fusion proteins tag, eYFP or myc (present on the BirA* constructs). Apoptosis was quantified by nuclear fragmentation. Data represents mean and SD of 3 independent experiments. Data analysed by one-way ANOVA, followed by Tukey’s multiple comparison test. **** represents p <0.0001. Scale bar = 10µm.

(B) HEK293T cells transiently expressing tBid-BirA* or BirA* alone were incubated overnight in the presence or absence of 50 µM biotin. In the presence of biotin tBid-BirA* and BirA* are seen to self-label at the predicted molecular weights (indicated by red arrows). The signal from self-labelled tBid-BirA* appears weaker than BirA*- alone as tBid is a potent inducer of apoptosis. Thus, fewer tBid-BirA* positive cells remained viable after biotin-labelling compared to BirA*-alone. Endogenously biotinylated proteins are detected at around 280, 130, 76 and 74 kDa.

(C) HEK-293T cells were transfected with plasmids expressing either GFP-Bcl-XL, or GFP-Bcl-XL and tBid-BirA*, then incubated overnight in the presence of 50 µM biotin. Biotin labelled proteins were isolated from whole cell lysates (WCL) on streptavidin beads. The WCL, the unbound fraction and the streptavidin bead bound fraction were separated by SDS-PAGE and immunoblotted for either GFP or biotin.

(D) HEK-293T cells transfected and treated as in B with overnight culture in the presence or absence of 50 µM biotin as indicated. Cells were fixed and immunostained for the myc-tag and biotin. Scale bar = 10µm.

**Figure S2.** *Bid and VDAC2 co-localizes at the mitochondria.*

(A) MCF-7 cells transiently expressing VDAC2-V5 were immunostained with anti-V5 and imaged using widefield microscopy.

(B) MCF-7 cells stably expressing BidWT-GFP were transiently transfected with a VDAC2-V5 expression plasmid. Cells were immunostained for GFP and V5 and imaged using widefield microscopy

(C) Cells as in (B) imaged by confocal microscopy followed by image deconvolution (B).­ In all panels, scale bars represent 10µm.

**Figure S3**. *Human breast cell lines display heterogeneous responses to Taxol induced prolonged mitosis.*

Examples of time-lapse sequences representing (A) Normal division (B) Apoptosis in mitosis (C) Mitotic slippage.

(D) Single cell fate profiles of MCF-7 and MDA-MB-231 cell lines treated with either vehicle only (control) or with 1μM Taxol over a 65 hour period. Data represents 90 cells tracked over three independent repeats.

**Figure S4.** *Phosphorylation of Bid regulates mitotic apoptotic priming in MCF7 breast cancer cells.*

(A) Quantification of hBid knock down and mBid re-expression. Left hand panel - pixel intensity was quantified using proteins detected with IRDye secondary antibodies and an Odyssey CLx imager (LiCor). Quantification of blots was performed using ImageStudio (LiCor) for bands relating to hBid and mBid in WT and mBidWT-GFP lanes. h/mBid band pixel intensities were normalised to vinculin loading control (Fig.4A). Right hand panel - with the addition of shBid, hBid is knocked down to 35% of endogenous proteins levels. Re-expression of exogenous mBid is 45% of endogenous protein levels. The total expression of Bid (combined expression of hBid and mBid) in mBidWT-GFP cells is approximately 80% of WT MCF-7. Data represents two independent experiments. Mean and SD are displayed.

(B) Duration of time modified MCF-7 cells remained in mitosis after treatment with taxol, separated by fate. Right hand panel displays data from cells that underwent mitotic slippage only, whereas left hand panel displays data from cells that underwent apoptosis only. Data derived from (4C), mean and SD plotted.

**Figure S5.**  *Phosphorylation of Bid regulates apoptotic priming in mitotic breast cancer cells.*

(A) Single cell fate profiles of the MCF-7 lines, untreated or treated with 1μM Taxol, in the presence of 5μM ABT-737 over 50 hours. Data represent 90 cells tracked over 3 independent repeats.

(B) Summary of apoptosis in mitosis for the data in (A) for the indicated MCF-7 cell lines. Mean and SD are shown. Data were analysed by one-way ANOVA, followed by Tukey’s multiple comparison test (ns = non-significant; * = p<0.05; ** = p<0.01).

(C) Single cell fate profiles of modified MDA-MB-231 lines treated with/without 1μM Taxol and/or 5μM ABT-737 over a 50-hour period. Data represents 90 cells tracked over 3 independent repeats.

(C) Summary of apoptosis after 50 hours for the data in (C). Data were analysed by one-way ANOVA, followed by Tukey’s multiple comparison test (ns = non-significant).

**Figure S6.** VDAC2 coordinates Bid phosphorylation dependent apoptotic priming in mitosis.

(A) Single cell fate analysis of D11 VDAC2 KO MCF-7 cell lines stably expressing VDAC2 V5, shBid alone or shBid in conjunction with the indicated mouse Bid-GFP variant (BidWT-GFP, BidS66A-GFP, BidG94E-GFP). Cells were untreated or treated with 1μM taxol and/or 5 μM ABT-737 over 48 hours. Data represents 90 cells tracked over 3 independent experiments.

(B) Lysates from unsynchronized WT MCF-7, D11 (VDAC2-ko) and D11 + VDAC2-V5 cell lines were analysed by immunoblotting, probing with antibodies against: Mcl-1, Bcl-XL, Bak, Bim, Puma, hBid and Bax. Anti-vinculin serves as a loading control.
