# Supplementary figures and images for: BioID-based proteomic analysis of the Bid interactome identifies novel proteins involved in cell-cycle-dependent apoptotic priming
Source: Cell Death Dis. 2020 Oct 16;11(10):872. doi: 10.1038/s41419-020-03091-8 (PMC7567853; doi:10.1038/s41419-020-03091-8)

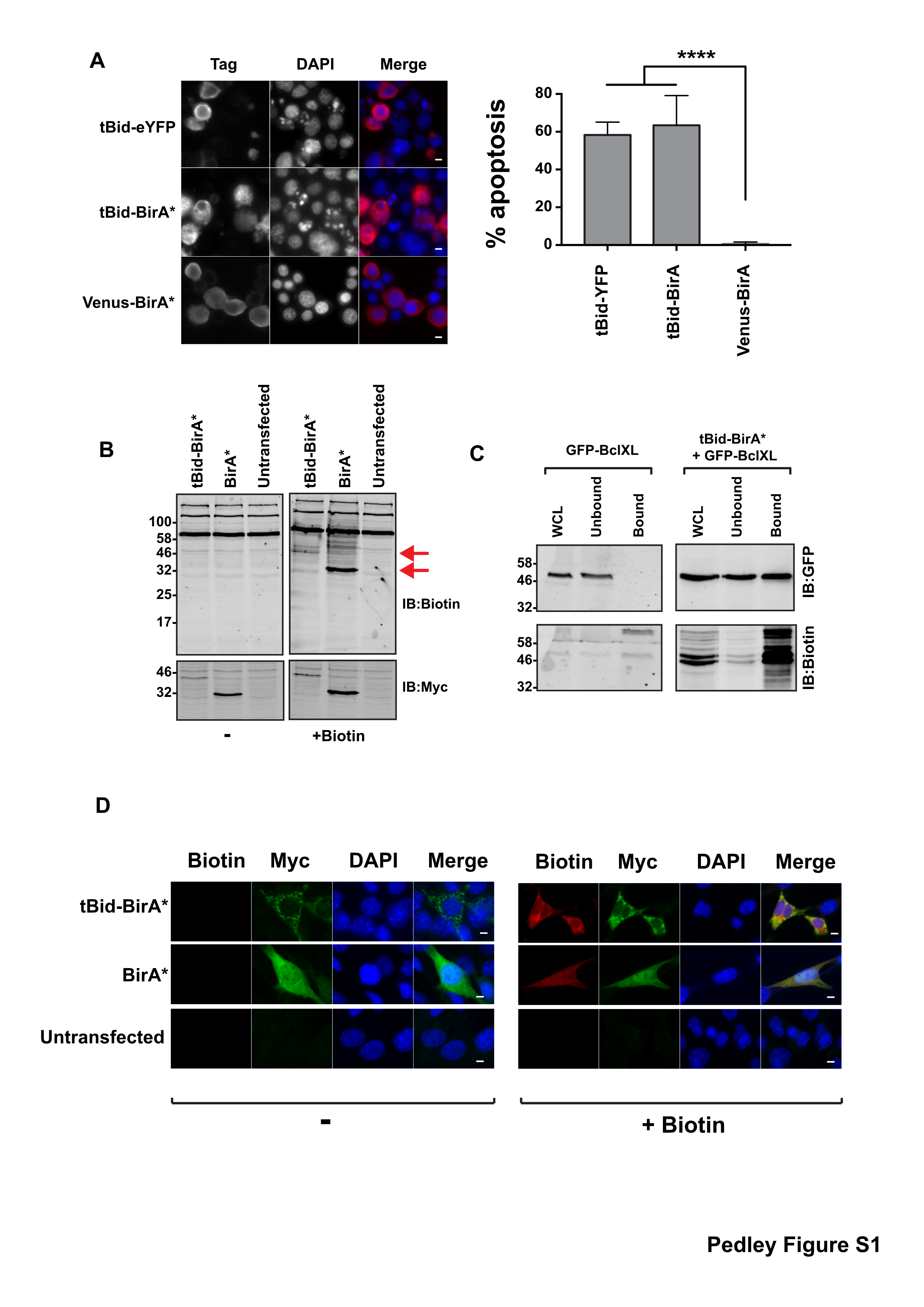

Supplement: Supplementary file 2 — supplementary figure 1 [file 41419_2020_3091_MOESM2_ESM.tif]

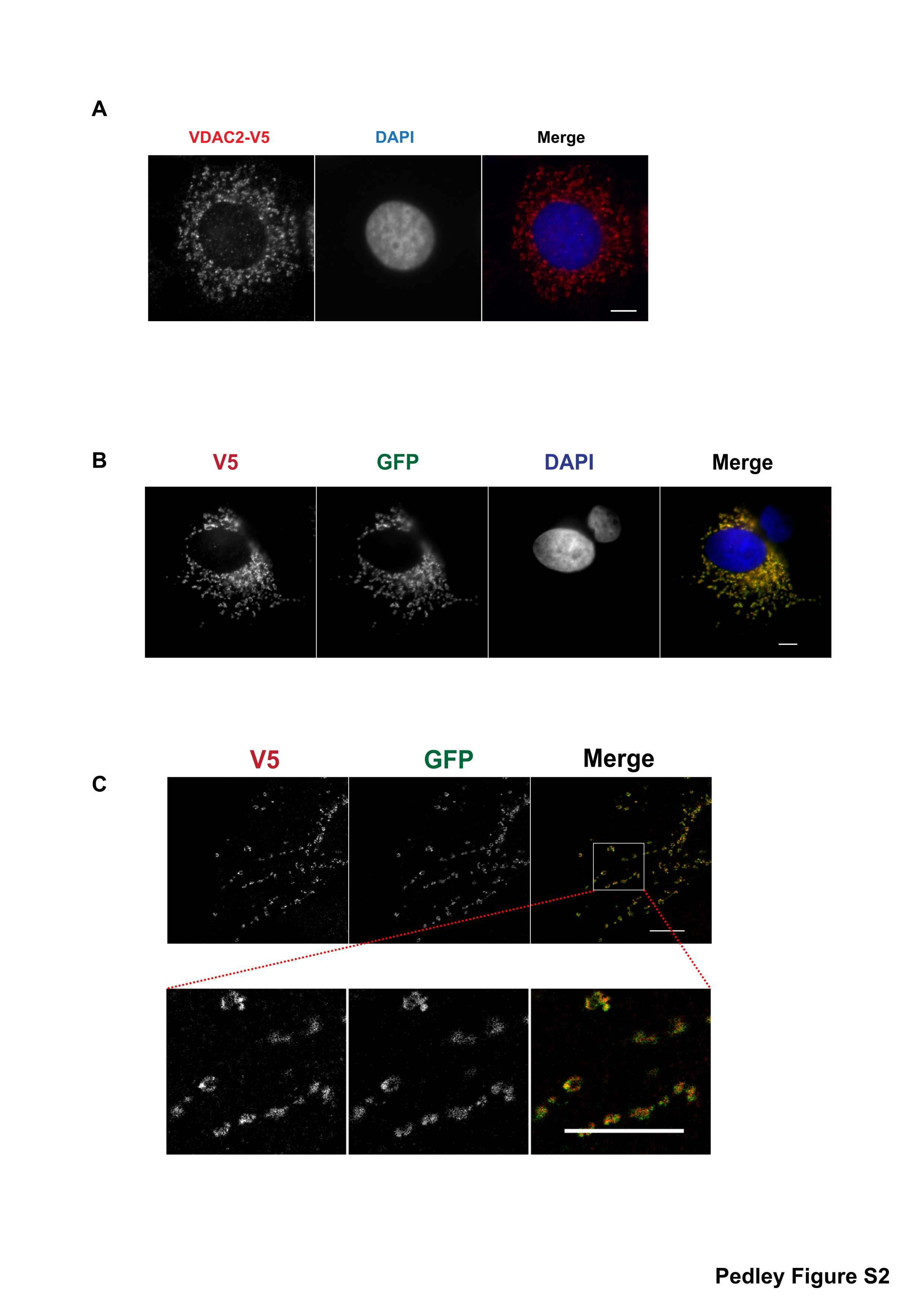

Supplement: Supplementary file 3 — supplementary figure 2 [file 41419_2020_3091_MOESM3_ESM.tif]

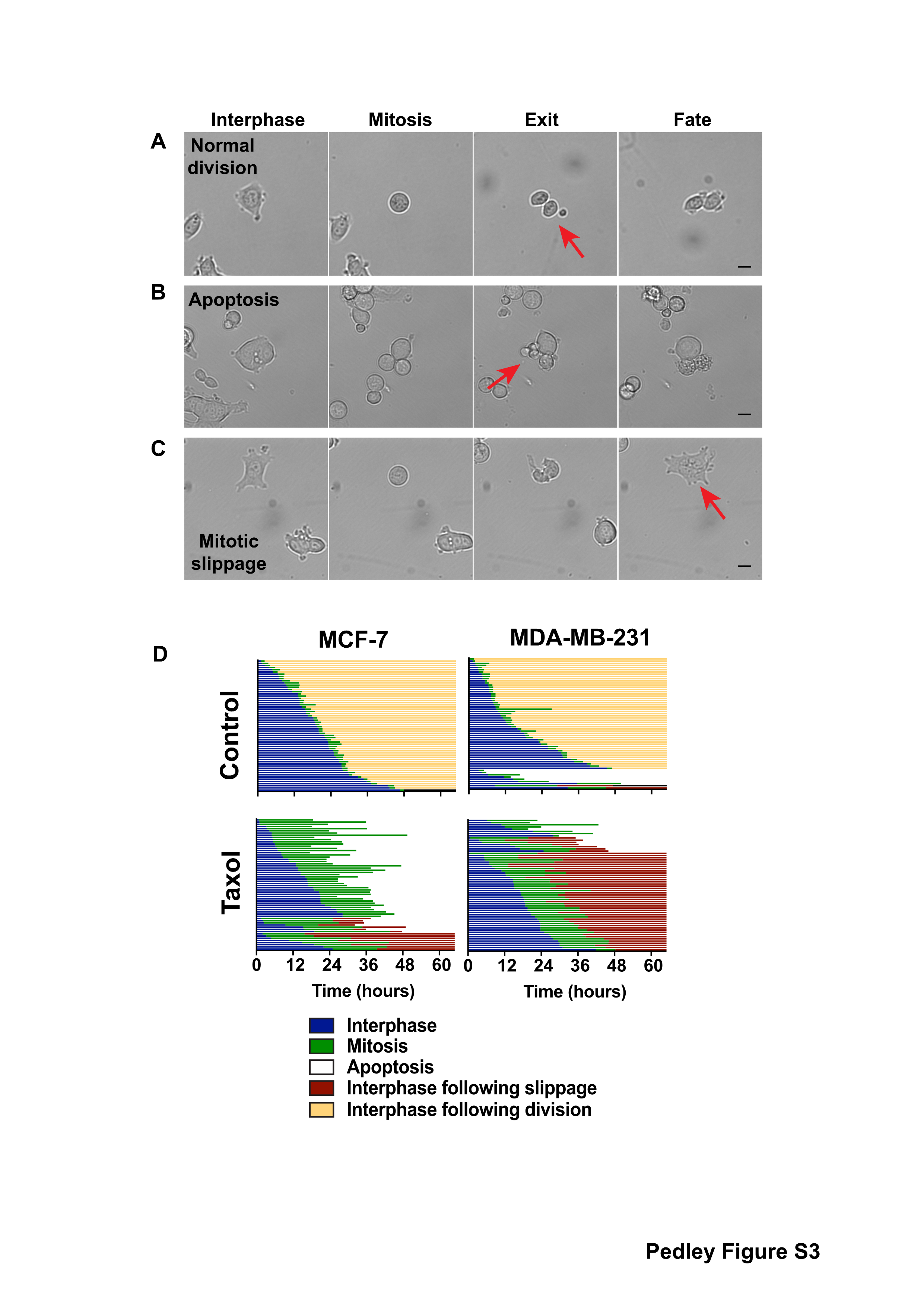

Supplement: Supplementary file 4 — supplementary figure 3 [file 41419_2020_3091_MOESM4_ESM.tif]

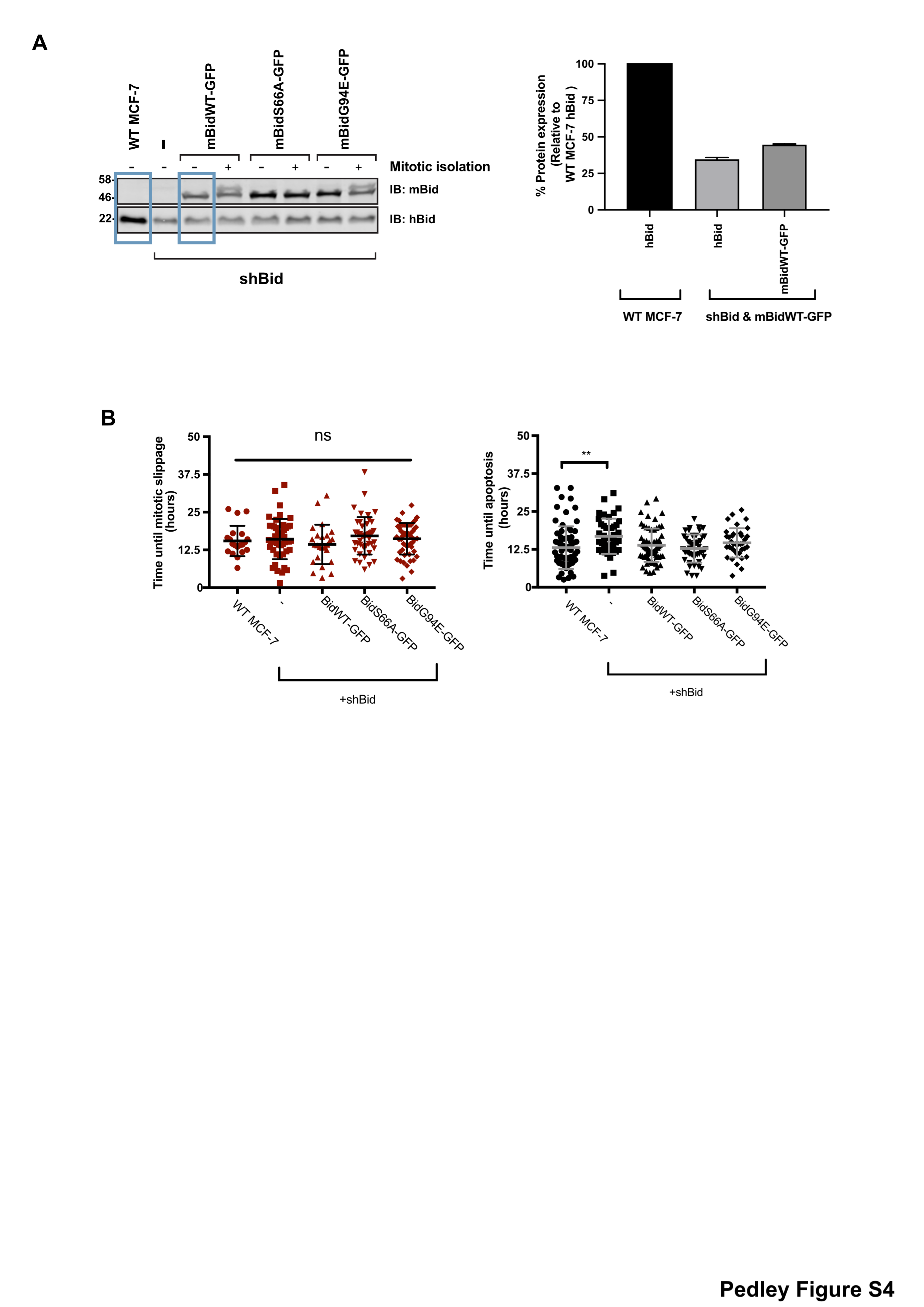

Supplement: Supplementary file 5 — supplementary figure 4 [file 41419_2020_3091_MOESM5_ESM.tif]

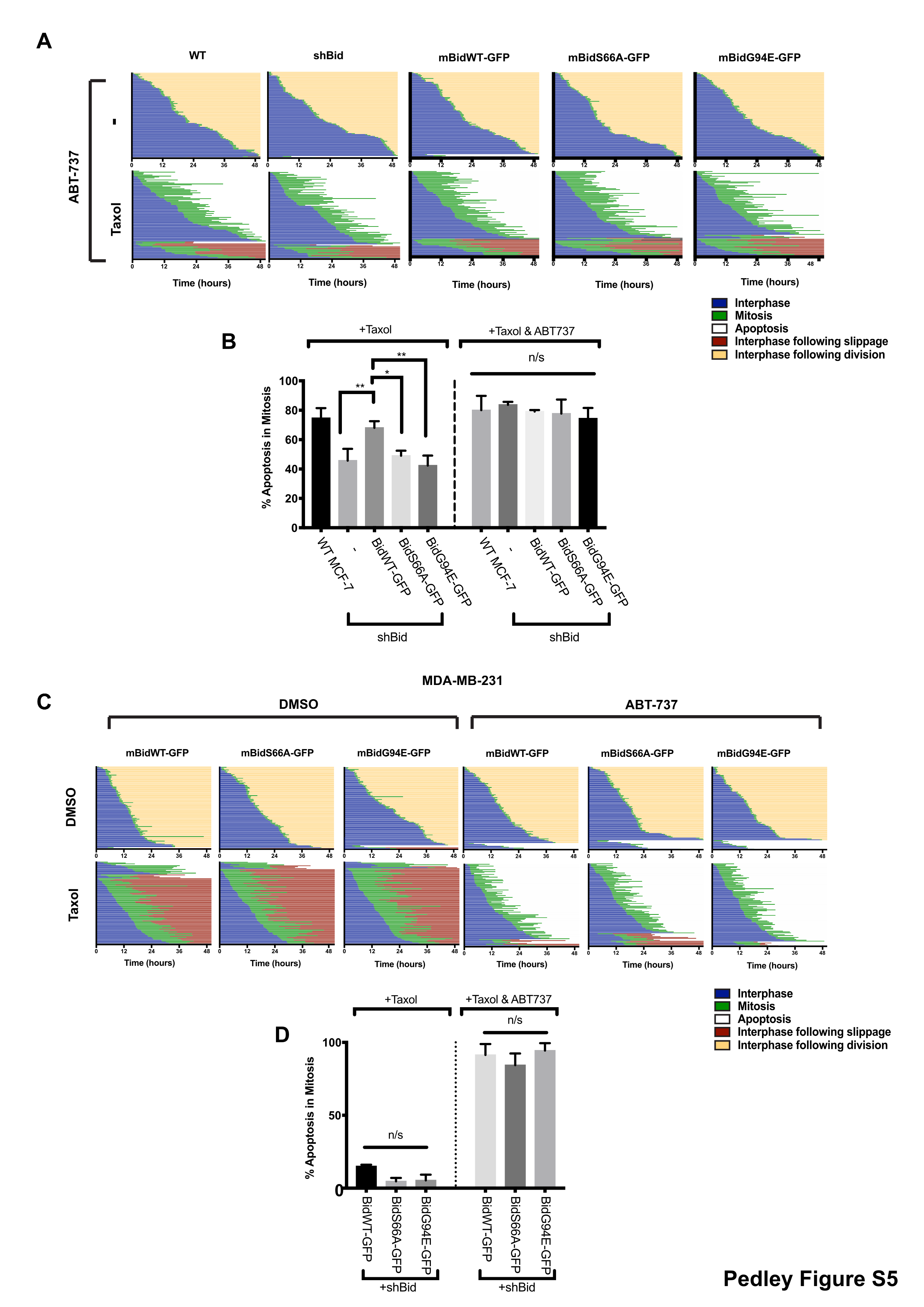

Supplement: Supplementary file 6 — supplementary figure 5 [file 41419_2020_3091_MOESM6_ESM.tif]

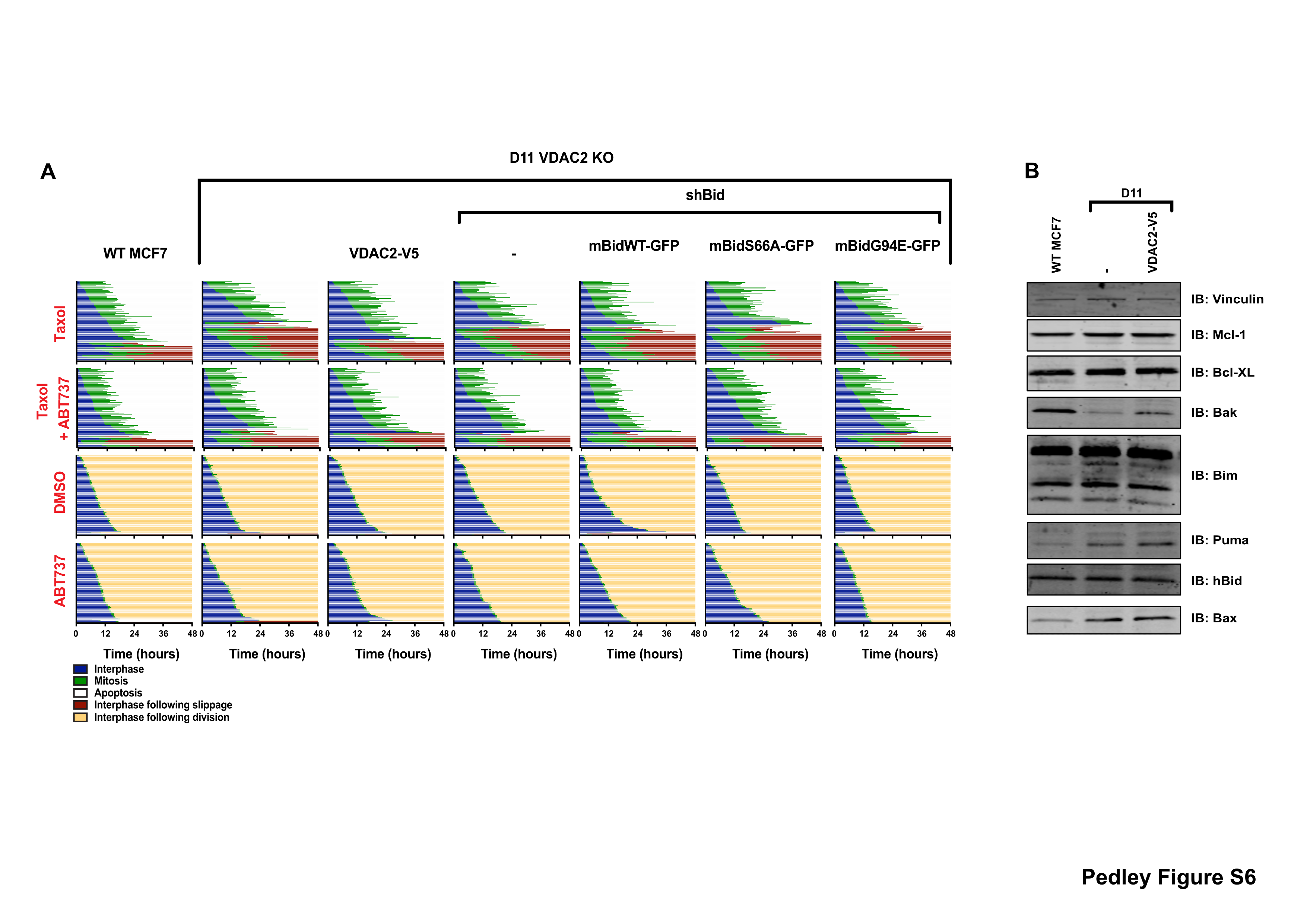

Supplement: Supplementary file 7 — supplementary figure 6 [file 41419_2020_3091_MOESM7_ESM.tif]
